# Barriers to genetic manipulation of Enterococci: Current Approaches and Future Directions

**DOI:** 10.1093/femsre/fuac036

**Published:** 2022-07-26

**Authors:** Alexandra L Krause, Timothy P Stinear, Ian R Monk

**Affiliations:** Department of Microbiology and Immunology, The University of Melbourne at the Peter Doherty Institute for Infection & Immunity, Melbourne, VIC 3000 Australia; Department of Microbiology and Immunology, The University of Melbourne at the Peter Doherty Institute for Infection & Immunity, Melbourne, VIC 3000 Australia; Department of Microbiology and Immunology, The University of Melbourne at the Peter Doherty Institute for Infection & Immunity, Melbourne, VIC 3000 Australia

**Keywords:** *Enterococcus faecium*, *Enterococcus faecalis*, restriction modification, electroporation, molecular biology

## Abstract

*Enterococcus faecalis* and *Enterococcus faecium* are Gram-positive commensal gut bacteria that can also cause fatal infections. To study clinically relevant multi-drug resistant *E. faecalis and E. faecium* strains, methods are needed to overcome physical (thick cell wall) and enzymatic barriers that limit the transfer of foreign DNA and thus prevent facile genetic manipulation. Enzymatic barriers to DNA uptake identified in *E. faecalis* and *E. faecium* include type I, II and IV restriction modification systems and CRISPR-Cas. This review examines *E. faecalis* and *E. faecium* DNA defence systems and the methods with potential to overcome these barriers. DNA defence system bypass will allow the application of innovative genetic techniques to expedite molecular-level understanding of these important, but somewhat neglected, pathogens.

## Teaser

The molecular biology of *Enterococcus faecalis* and *Enterococcus faecium* has lagged behind other Gram-positive pathogens due to physical and enzymatic barriers restricting DNA transfer, with this review summarising the barriers and highlighting how improving transformation will accelerate our understanding of these important pathogens.

## Introduction

The Gram-positive genus *Enterococcus* comprises over 50 species of commensal gastrointestinal (GI) bacteria that make up about 1% of the total microbial load in healthy individuals, but some species of the genus are also a major cause of nosocomial infections (causing 14% of all hospital acquired infections in the US between 2011 and 2014) (Fiore et al.2019a). They can inhabit various niches including different regions of the GI tract and oral cavity of humans and other animals, but they can also be recovered from water, soil, plants and processed food (Franz et al. 2003, Byappanahalli et al. 2012, Hammerum 2012, Lebreton et al. 2014, Komiyama et al. 2016, Dubin et al. 2017). The most notable enterococcal human pathogens are *Enterococcus faecalis* and *Enterococcus faecium*. When pathogenic enterococci gain access to niches in the human body beyond their commensal habitat of the GI tract, such as the urinary tract, skin (via wounds/burns), bloodstream and the heart, they have a high potential to cause persistent infections and subsequent serious disease (Fiore et al.2019a). These types of infections are more common in people with underlying risk factors that include immunosuppression, renal/liver disease, diabetes mellitus, or abdominal and oral surgery (Billington et al. 2014, Kajihara et al. 2015).

Enterococcal infections, in particular those caused by *E. faecalis* and *E. faecium*, can be very problematic because of the propensity of the bacteria for intrinsic and acquired multiple drug resistance (MDR). *E.faecium* infections can be very difficult to treat because of high levels of ampicillin and vancomycin resistance, compared to *E. faecalis* where ampicillin and vancomycin resistance are less frequently reported. Antibiotics such as linezolid, daptomycin, tigecycline, and quinupristin-dalfopristin are commonly used to treat MDR enterococcal infections, however, resistance to these drugs has also been observed (Kristich et al. 2014, Mercuro et al. 2018). Analysis of *E. faecium* clinical isolates found that 13.4% (106/791) compared with 1.4% (9/651) *E. faecalis* strains were resistant to linezolid (Malisova et al. 2021). A cohort study collected *E. faecium* isolates from patients colonised with vancomycin resistant enterococci (VRE) and found that 11.7% (50/426) of the strains examined were also resistant to daptomycin (Kinnear et al. 2020), observations that are in line with a resistance rate (15%–49/324) recently reported from a collection of Australian and New Zealand *E. faecium* isolates (Li et al. 2021). *E. faecalis* is intrinsically resistant to quinupristin-dalfopristin conferred by the *lsa* gene, with resistance in *E. faecium* also mediated by drug efflux (Hollenbeck and Rice 2012). *E. faecium* resistance to quinupristin-dalfopristin was 1% (9/911) in a study from China (Wang et al. 2016), but 9.4% (16/170) in Australia (Coombs et al. 2013). Tigecycline resistance is reported to only be 1% for *E. faecium* and 0.3% for *E. faecalis*, which might be due to the bacteriostatic mode of action of the drug (Dadashi et al. 2021). In agriculture, the use of antibiotics including tetracycline, streptomycin, avoparcin (a glycopeptide) and phenicols (often though co-location on the chromosome with *optrA* or *cfr* conferring linezolid resistance) are a major driver of antibiotic resistance in enterococci (Manson et al. 2004, Kristich et al. 2014, Nuesch-Inderbinen et al. 2021, Timmermans et al. 2021). To combat the MDR problem, newer antibiotics such as dalbavancin, oritavancin, tedizolide, and telavancin are being used but with varying degrees of success and availability (Kristich et al. 2014, Esmail et al. 2019, Krawczyk et al.^2021^).

When the GI tract of a patient is colonised with VRE, the use of pre- (or post) operative antibiotics can cause VRE to become the dominant species in the gut microbiota and then spread to other body sites (Fiore et al. 2019b, Krawczyk et al.^2021^). MDR strains of VRE are an increasing burden on the healthcare system. Transient carriage of MDR VRE on the hands of healthcare workers can be a major transmission vector and increase hospital infections if not properly cleaned and sanitised (Grayson et al. 2012, Agudelo Higuita and Huycke 2014, Krawczyk et al.^2021^) with some *E. faecium* strains also exhibiting an increased tolerance to alcohol (Pidot et al. 2018).

*E. faecium* strains can be divided into subspecies made up of clades A and B, typically clade A are MDR *E. faecium* strains and clade B are commensal, non-hospital, antibiotic susceptible strains, which have been recently renamed *E. lactis*. (Belloso Daza et al. 2021). A further subspecies divergence has been identified among the clades into A1 and A2. Clade A1 are linked to more infective, endemic hospital strains, while clade A2 are linked to periodic infection in humans from an animal reservoir (Lebreton et al. 2013). With increased genomic resolution afforded through the analysis of a larger *E. faecium* strain collection from the UK and Ireland, the original A1/A2 separation was not supported. However, A2 clade isolates were shown to be ancestorial to isolates from A1 (Raven et al. 2016). A further in-depth analysis of a representative collection of international *E. faecium* isolates, suggested that a continuum exists between the A and B clades, with A comprising the dominant hospital lineage (van Hal et al. 2022). Further subgroups, approximately equivalent to the clade structure of A1 and A2, identified by Lebreton et al, could be distinguished (Lebreton et al. 2013). The authors suggest that these can be missed if pre-hospital colonising (predominantly A2 and B) and hospital acquired isolates (A1) are not adequately sampled. *E. faecium* is highly recombinogenic, with gene flow observed from A2 or B isolates to A1 isolates. Each group contains a unique repertoire of genes allowing for the generation of enhanced genetic diversity through gene introduction or formation of hybrid genomes (van Hal et al. 2022). Additionally, *E. faecium* contains extensive diversity in their plasmid content, with the complete plasmidome of 62 clade A isolates recently described, showing the presence of 305 plasmids. Interestingly, *E. faecium* isolates from hospitalised patients contained a significantly increased plasmid DNA content. Optimal plasmid configurations were potentially important in improving strain fitness in the hospital environment (Arredondo-Alonso et al. 2020). In contrast, limited phylogenetic distribution has been observed in *E. faecalis*, without an obvious lineage or clade structure (Palmer et al. 2012).

The presence of restriction modification (RM) systems leading to host specific DNA methylation signatures are major hurdles that prevent detailed molecular studies of these opportunistic pathogens. Most of the fundamental research on the biology and pathogenesis of *E. faecalis* and *E. faecium* has been conducted with select strains able to be transformed efficiently with plasmids isolated from *Escherichia coli* (Jacob and Hobbs 1974, Paulsen et al. 2003, Nallapareddy et al. 2006b, Bourgogne et al. 2008, Zhang et al. 2012). However, these strains are not representative of most clinical isolates (Nallapareddy et al.2006a, Huo et al. 2015, de Maat et al. 2020), where physical and RM barriers prevent the introduction of foreign DNA. The inability to readily and reliably introduce DNA, to conduct targeted or random mutagenesis in *E. faecalis* and *E. faecium* represents a major bottleneck for research efforts to understand their pathobiology. In this review, we will detail the physical and enzymatic barriers present on the outside/inside of the enterococcal cell, respectively, with a specific focus on *E. faecium*. Progress in breaching these barriers will facilitate the application of new genetic tools and accelerate progress towards an improved understanding of *E. faecium* at a molecular level.

## Gram-positive cell wall structure; a barrier to foreign DNA uptake

**Figure 1.**
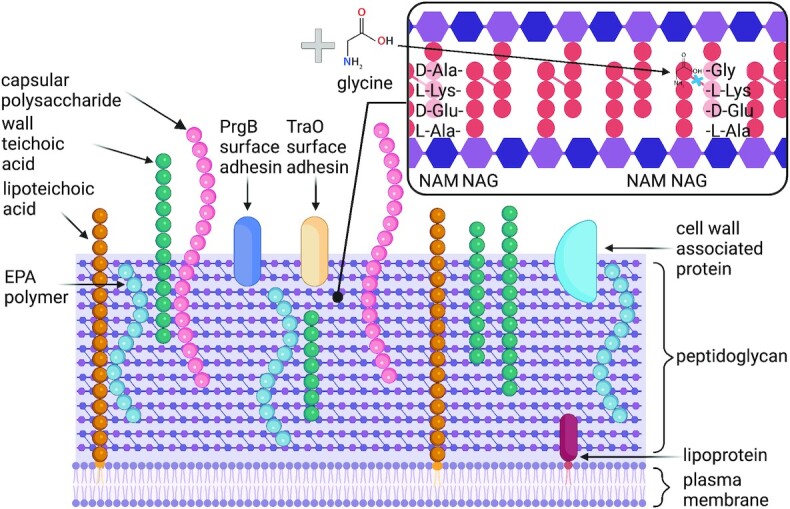
The enterococcal cell wall architecture. The cell wall of enterococci is composed of a thick peptidoglycan cross-linked lattice of repeating sugars (N-acetylmuramic acid (NAM) and N-acetylglucosamine (NAG)) containing wall teichoic acids, lipoteichoic acids, capsular polysaccharides and enterococcal polysaccharide antigen (EPA) polymers surrounding a plasma membrane made up of a phospholipid bilayer. The cell wall can also contain membrane bound (lipoprotein shown) or cell wall bound proteins (sortase A linked PrgB and TraO surface adhesions are shown). The impact of glycine overload on peptidoglycan crosslinking is detailed (Shockman and Barrett 1983, Hancock et al. 2014, Brown et al. 2015). Created with BioRender.com.

The thick enterococcal cell wall (approximately 40 nm) is composed of peptidoglycan (PG), interspersed with wall polymers such as wall teichoic acid (WTA) or other polysaccharides. Lipoteichoic acids (LTA) extend from the top of the peptidoglycan layer and is anchored to the plasma membrane by covalent bonds (Fig. 1) (Shockman and Barrett 1983, Hancock et al. 2014, Brown et al. 2015, Yang et al. 2017, Chang et al. 2018). The cell wall also contains cell wall polysaccharides such as capsular polysaccharides (Cps) which are embedded in the cell wall and extend outwards, as well as enterococcal polysaccharide antigens (EPA) (Hancock et al. 2014).

The recent in-depth review from Ramos et al. summarises the roles of enterococcal surface polysaccharides in the host-pathogen interaction (Ramos et al. 2021). The main component of the cell wall is PG and this forms a rigid repeating lattice structure of glycans (N-acetylmuramic acid and N-acetylglucosamine). These sugars are linked together by glycosidic bonds catalysed by a transglycosylase. (Shockman and Barrett 1983). Schleifer and Kilpper-Balz (Schleifer and Kilpper-Bälz 1987) showed that of 7 out of 8 enterococcal species examined contain a peptide cross bridge comprising of a single D-Asp, whereas in *E. faecalis* it is comprised of 2–3 L-Ala residues. In *E. faecium* cross bridge location on the linking residues can be altered by environmental conditions (Ampicillin or Vancomycin exposure) and genetic background (VSE vs VRE). The substrate for penicillin binding proteins (D,D-transpeptidase) is the pentapeptide stem, which catalyses 4 (D-Ala)-3 (L-Lys) crossbridge (D-Asp or L-Ala^2-3^ formation). Alternately, in the presence of tetrapeptide substrate which can occur through two independent mechanisms depending on the antibiotic, a L,D-transpeptidase can catalyse 3(L-Lys)-3(L-Lys) cross-bridge formation (Pidgeon et al. 2019). These strong bonds unify the PG in the cell wall making it very hard to penetrate, even with electroporation, without first weakening the cell wall to get DNA in the cell (Aune and Aachmann 2010). Glycine added to growth media is commonly used to weaken the cell wall of Gram-positive bacteria as it can substitute a L-alanine or D-alanine in the stem peptide (Fig. 1) that subsequently reduces the strength of the cross linking (Hammes et al. 1973). Cell wall weakening occurs as the altered glycine-containing PG precursors are often not cross-linked by a transpeptidase, which can lead to changes in cell morphology (Hammes et al. 1973, Bhattacharjee and Sorg 2020). This approach has been successfully applied to the generation of electrocompetent cells in a wide array of Gram-positive bacteria (Holo and Nes 1989, Buckley et al. 1999, Turgeon et al. 2006, van Pijkeren et al. 2010, Chang et al. 2017, Bhattacharjee and Sorg 2020). Alterations to the terminal pentapeptide stem residues from D-Ala-D-Ala to D-Ala-D-Lac in VRE confers vancomycin resistance due to reduced target binding affinity (Hancock et al. 2014), while the impact of this cell wall modification on transformation has not been determined. Overall, the thick rigid lattice structure of the peptidoglycan layer in the cell wall poses a substantial barrier to DNA uptake without any other mechanism of natural DNA competence present in enterococci.

A second principle component of the cell wall are the essential teichoic acids, of which there are two types of anionic glycopolymers; WTA and LTA (Theilacker et al. 2012). In enterococci teichoic acids have roles in host cell interaction, biofilm formation, resistance to antimicrobial peptides and also cell wall cation homeostasis (Theilacker et al. 2012, Ramos et al. 2021). In Gram-positive bacteria the *tagO* (also called *tarO*) gene is the first committed step of WTA biosynthesis (Holland et al. 2011, Palmer et al. 2012, Brown et al. 2013, Winstel et al. 2013) with the enterococcal ortholog being *epaA* (Palmer et al. 2012). In *S. aureus*, in addition to other roles, WTA acts as temporal and spatial regulator of peptidoglycan metabolism (Atilano et al. 2010). Deletion of *tagO* has pleiotropic effects (impaired cell division, reduced PG cross-linking, protein localisation and virulence) in all bacteria examined, with the deletion from *Staphylococcus epidermidis* also enhancing transformation by electroporation, indicating a potential role of WTA composition in limiting horizontal gene transfer (HGT) (Atilano et al. 2010, Holland et al. 2011). WTA serves as a receptor for certain *Staphylococcus aureus* phages, variation in WTA glycosylation can impact on phage host range (Winstel et al. 2013).

The presence of a capsule has been shown to reduce DNA uptake in a number of bacterial pathogens. In *E. faecalis* it plays a role in host immune evasion and resistance to opsonophagocytosis (Fournet-Fayard et al. 1995, Jeon et al. 2009, Thurlow et al. 2009, Brown et al. 2013). Additionally, mutations in capsule biosynthesis are likely to have an impact on the integrity of the enterococcal cell wall, which could be beneficial for transformation (Ali et al. 2019). EPA is another conserved enterococcal cell wall antigen which is composed of repeating sugar subunits that can vary between enterococcal strains and has been shown to be involved in immune evasion, intestinal colonisation and virulence (Chatterjee et al. 2019, Smith et al. 2019). When EPA is mutated, it is shown to decrease peptidoglycan crosslinking and impair cell wall integrity (Smith et al. 2019). Currently, the impact of EPA on enterococcal electroporation is unknown, but it plays an essential role in the adsorption and infection by lytic bacteriophages in *E. faecium* and *E. faecalis* (Chatterjee et al. 2019, Canfield and Duerkop 2020, Canfield et al. 2021).

A number of publications have systematically analysed the cell wall as a barrier in specific Gram-positive bacteria to develop optimal treatments to achieve maximal DNA transfer by electroporation (Aune and Aachmann 2010). While the cell wall of *E. faecalis* and *E. faecium* provides a formidable barrier, strains can also encode a strong second line of defence, as discussed below (Manso et al. 2014, Lee et al. 2019).

## DNA defence systems in *E. faecium* and *E. faecalis*

The transfer of DNA between bacteria encourages evolution by the introduction and incorporation of new genetic material. However, there is a balance between essential and superfluous genes and the need for bacteria to maintain a streamlined genome to be competitive in complex microbiomes (Oliveira et al. 2014). There are two main bacterial defense mechanisms that prevent HGT, either clustered regularly interspaced short palindromic repeats and the associated proteins (CRISPR-Cas) (Nidhi et al. 2021) or RM systems (Murray 2000).

CRISPR-Cas function as a bacterial ‘immune system’ that evolves through integration of DNA recognised as foreign into a CRISPR array, which then become CRISPR guide RNAs. The guides target foreign DNA to be specifically degraded by nuclease encoded activity. The presence of CRISPR-Cas is generally more common in commensal enterococcal strains which prevent the uptake of foreign DNA such as the acquisition of drug resistance elements (Johnson et al. 2021). Conversely, MDR/clinical enterococcal strains either lack these defence systems or contain non-functional variants such as CRISPR2 (contains spacer array but no *cas* genes) (Johnson et al. 2021). Therefore, the MDR strains have increased genetic plasticity which can enhance survival compared to strains that lack genetic diversity due to the absence of MDR genes (Johnson et al. 2021). This is exemplified in *E. faecium*, where in clade B strains (commensal strains) examined, CRISPR-Cas were sporadically present, with few MGE. While clades A1/A2, no CRISPR-Cas genes were identified and the genome were littered with antimicrobial resistance (AMR) determinants (Lebreton et al. 2013). However, this simplistic view does not take into account the presence of other barriers discussed below. A recent comprehensive genomic analysis of all available genome sequences identified a greater prevalence of CRISPR spacers in *E. faecalis* strains (∼75%) compared to ∼5% in *E. faecium* strains (Mlaga et al. 2021), but this did not assess whether the putative systems identified were functional.

The second defence mechanism is RM, with only three out of the four known RM systems described in *E. faecium* and *E. faecalis* (the type III RM systems have not been detected).

Type I RM systems are the most complex and are made up of three protein subunits, termed host specificity of DNA (Hsd), specificity (S) protein, the modification (HsdM) (methylation) protein and the restriction endonuclease (HsdR) protein (Monk and Foster 2012). HsdM and HsdS form a protein complex (HsdM_2_ HsdS_1_) yielding the adenine methylase activity. The complex binds to a specific DNA sequence dictated by the two-target recognition domains (TRD) of the HsdS. These DNA motifs are comprised of an asymmetric bipartite DNA sequence with the two TRD DNA motifs comprising 3–4 base pairs (containing the methylated adenine residue) separated by 4 to 9 non-specific base pairs. In the presence of TRD specific adenine methylation (on hemi-methylated replicating DNA), methylation occurs at the corresponding unmethylated TRD site. The addition of two HsdR subunits to the HsdM_2_HsdS_1_ complex at completely unmethylated sites transforms the system into a molecular motor and it is translocated along flanking DNA via ATP hydrolysis, breaking when collision with either a second bound RM complex or DNA secondary structure. Therefore, Type I RM systems do not yield a defined cleavage sequence pattern. The formation of the restriction complex and subsequent DNA cleavage is inhibited by the correct adenine methylation profile.

Type II RM systems are a diverse collection of enzymes with sequence specific DNA cleavage, with examples including type IIP *EcoRI* or type IIS *BsaI*. Cleavage can be prevented or activated by the presence or absence of specific methylation on the recognition sequence. These enzymes have been the ‘workhorses’ in molecular biology, with their discovery and application have dramatically accelerated the application of recombinant DNA technologies in biotechnology (Roberts 2005). A comprehensive review of type II RM systems was published in 2014 (Pingoud et al. 2014).

Type IV RM systems are the simplest and function by restriction enzyme cleavage of modified DNA e.g. methylated or glycosylated DNA. They lack methyltransferase activity, with the system operating inversely to the other RM systems (Loenen et al. 2014). Type IV systems have been identified across a broad array of Gram-positive and Gram-negative bacteria, where they present a major barrier to prevent foreign DNA uptake (Gonzalez-Ceron et al. 2009, Xu et al. 2011, Sitaraman 2016, Gifford et al. 2019, Woods et al. 2019, Picton et al. 2021).

PacBio (a DNA sequencing platform that can specifically discriminate methylated DNA bases) sequencing (Flusberg et al. 2010, Clark et al. 2012) has helped identify the contribution of RM in *E. faecium* and *E. faecalis* to the development of the distinct genetic clades (Furmanek-Blaszk and Sektas 2015, Huo et al. 2015). Sequencing confirms that clinical isolates like *E. faecium* strains 6E6 and 2014-VREF-63 have 6-methyl adenine DNA methylation, which is associated with a type I RM system and 5-methyl cytosine, which is associated with a type II RM system (Huo *et al*. 2019).

*E. faecium* isolates also harbour a type IV RM system that is overrepresented in B and A2 clades (Lebreton et al. 2013), while clade A1 clade strains are enriched for a specific type I RM system (Huo et al. 2019). It has been suggested that these DNA defence systems could be a driver for the formation of distinct clades found in *E. faecium* and potentially contributing to the issues with genetic manipulation of these strains (Lebreton et al. 2013). Comparative genomic analysis of 73 *E. faecium* strains (15 clade B, 21 clade A1, 35 clade A2 and 2 hybrid isolates) identified a conserved type I system present in clade A1, with specific *hsdM* and *hsdR* alleles identified in 18/21 isolates (Lebreton et al. 2013). A single HsdS allele was found in 14/21 clade A1 isolates, which may have contributed to A1 subspeciation and selected for phenotypes with improved survival characteristics in healthcare settings. However, unlike in *S. aureus*, where up to 100% of a clonal complex contain a specific HsdS repertoire (Lee et al. 2019), the single type I system in clade A1 *E. faecium* might yield a partial barrier to DNA transfer. It will be important to screen more *E. faecium* genome sequences to assess the distribution of this particular *hsdS* allele. Interestingly, after deletion of the type I RM system (ΔRM) from two different *E. faecium* backgrounds (clade A1; strain 1231 502 and clade B; strain 1141 703) there was only up to a half log increase in transformation efficiency between the ∆RM mutant compared to the wild type (Huo et al. 2019). This questions the impact this RM system has on foreign DNA transfer. However, the test plasmid used for the electroporations contained only one HsdS recognition sequence. A plasmid containing multiple recognition sequences would help improve the resolution and clarify the impact of type I RM in limiting DNA transfer. A more recent take on the presence of type I RM related to plasmid content identified the same HsdS allele described by Huo et al (Huo et al. 2019) as being enriched, but also described an additional eight HsdS alleles (Arredondo-Alonso et al. 2020). Of these eight, four were found in A1 and two in non-A1 isolates. Authors suggest that type I RM may play a role in shaping the plasmid content of *E. faecium* clones through the restriction of DNA transfer (Arredondo-Alonso et al. 2020).

In *E. faecalis* type I RM systems are also present and can be found as phasevarions (phase-variable regulons); an epigenetic regulatory system (Atack et al. 2020a). Phase variation is the random switching on and off of gene expression at a high frequency, this typically occurs in genes encoding surface proteins such as pili or adhesins of host-adapted pathogens (Phillips et al. 2019a). The presence of a potential RM system in *E. faecalis* was first postulated in 1978 after plasmid DNA isolated from *E. faecalis* had a lower transformation efficiency compared to DNA isolated from *S. sanguis*. (Leblanc et al. 1978). The SfeI a type II RM system subsequently discovered in *E. faecalis*, is closely related to an ortholog from *Lactococcus lactis* (Okhapkina et al. 2002). Recently, two more type II RM systems have been discovered in *E. faecalis* OG1RF and *E. faecalis* NEB215 (Huo et al. 2015).

Phase variation can occur through simple sequence repeats (SSRs) located in an open reading frame or promoter region, leading to frameshift mutations or an impact on the expression level, respectively. Additionally, SSRs can be affected by length of the SSRs which can cause fluctuation in gene expression levels. Phase variation of type I RM through modulation of methyltransferase *hsdM* expression can lead to methylation differences across the genome resulting in a global impact on gene expression (Atack et al. 2020a,b). It was determined that around 10% of type I RM systems contain SSRs and can undergo phase variation of gene expression (Atack et al. 2020a). Invertase mediated recombination through TRD of co-localised HsdS alleles can change the specificity of the HsdS allele or inactivate the protein. When there are multiple HsdS proteins these can create complex phasevarions. These complex methylation patterns provide heterogeneity in gene expression which is akin to a bet-hedging strategy which can improve fitness under certain conditions (Reyes Ruiz et al. 2020).

The use of phase-regulated gene expression allows the bacteria to encode multiple genes that are beneficial for adapting to different environments e.g. evading immune response, colonisation of a different host niche or surviving in a harsh environment (Phillips et al. 2019a). Atack et al (Atack et al. 2020a), identified that 24/34 *E. faecalis* strains analysed contained a type I RM system. The 24 type I RM systems encoded multiple *hsdS* genes, with a high level of TRD variability. This showed that phase variable type I RM systems are present in *E. faecalis*, and begs the question if as yet unidentified phasevarions are also present in *E. faecium* (Phillips et al. 2019a, Huang et al. 2020, Atack et al. 2020a).

In *S. aureus*, both naturally occurring and constructed restriction deficient strains have been essential tools in unlocking *S. aureus* genetics (Kreiswirth et al. 1983, Monk and Foster 2012). These restriction deficient strains have the ability to accept large drug resistance plasmids from *E. faecalis* by conjugation, potentially contributing to the dissemination of vancomycin resistance genetic elements into *S. aureus* population (Sung and Lindsay 2007). This work highlighted that the restriction barrier can be overcome by using strains lacking fully functioning RM systems, whereby they are methylation proficient but restriction deficient. Further work has been conducted in *S. aureus* along these lines to streamline genetic manipulation. An *E. coli* strain, DC10B, was constructed through deletion of the *dcm* gene to prevent cytosine methylation of DNA and subsequent degradation by the conserved *S. aureus* type IV RM system (Xu et al. 2011). The combination of DC10B with an improved allelic exchange vector (pIMAY—pWV01ts and strong antibiotic selection) has streamlined construction of *S. aureus* and *S. epidermidis* mutants (Monk et al. 2012, Monk and Stinear 2021). Further *E. coli* and plasmid improvements have accelerated the ability to engineer *S. aureus* strains. Clonal complex specific type I RM methylation profiles were heterologously expressed from the chromosome of DC10B which allowed the complete bypass type I and type IV RM. The addition of blue/white screening into pIMAY (yielding pIMAY-Z) established phenotypic screening of plasmid loss (Monk et al. 2015). Similar plasmid modification techniques could be applied to *E. faecium* strains containing type I RM, as pIMAY-Z has already been successfully used in *E. faecium* (Pidot et al. 2018, Monk and Stinear 2021). We have found that the Prokaryotic Antiviral Defence Locator (https://padloc.otago.ac.nz/padloc/) bioinformatic tool as a rapid and user friendly method to assess the presence of RM systems and other barriers, which could impair plasmid transfer in enterococci (Payne et al. 2021).

## Horizontal gene transfer in enterococci

Certain bacteria such as *Haemophilus influenzae, Neisseria gonorrhoeae, Bacillus subtils*, and *Streptococcus pneumoniae* can actively uptake environmental DNA through the production type IV pili which pull DNA into the cell (Giltner et al. 2012, Mell and Redfield 2014, Muschiol et al. 2015). Under activating conditions cells become naturally competent with cues including cell density, presence of specific nutrients and induction of stress networks (Mell and Redfield 2014, Muschiol et al. 2015, Piepenbrink 2019). Type IV pili for DNA uptake have not been identified in enterococci. Other naturally occurring systems of DNA transformation and HGT are present in enterococci including conjugation and transduction, which are detailed below.

For bacteria which lack these natural competence systems, an ‘un-natural’ approach to DNA transformation is required to transfer recombinant DNA into the cell. One widely used approach involves the application of electric currents called electroporation, to induce transient pores in the bacteria cell wall and allow DNA to enter (Aune and Aachmann 2010). Prior to the development of electroporation, a common technique for HGT was protoplast transformation. PG was enzymatically digested which facilitated DNA uptake, with subsequent cell wall regeneration (Wirth et al. 1986). The natural HGT systems of conjugation and transduction can also be manipulated to allow for the targeted genetic modification.

## Conjugation

The transfer of DNA from a donor bacterial cell to a recipient cell which requires close contact and generation of a pilus/pore is called conjugation. This mechanism of transfer is common among plasmids and integrative conjugative elements (ICEs) harbouring AMR genes (Perry and Wright 2013, Kohler et al. 2019). However, in Gram-positive bacteria conjugation has not been extensively used as a tool for genetic manipulation as it relies on the bacteria expressing complex machinery to carry out conjugation (Type IV secretion system) and also has to overcome host enzymatic barriers such as CRISPR and RM (Dunny 2007, Strand et al. 2014). In *E. faecalis*, pheromone-inducible plasmid transfer is a common mechanism of plasmid transfer, with it less frequently observed in *E. faecium* (Sterling et al. 2020). This involves the secretion of pheromones by the recipient to the donor cells stimulating the production of aggregation proteins such as PrgB, which initiates close cell-to-cell contact, formation of a mating channel and then plasmid transfer (Clewell et al. 1985, Dunny et al. 1995, Sterling et al. 2020). Enterococci share ssDNA efficiently via conjugation. Conjugative plasmids can encode anti-restriction genes, like *ardA*. ArdA mimics dsDNA and inhibits type I RM activity allowing ssDNA to dsDNA conversion. (Marcinek et al. 1998, Murray 2000, McMahon et al. 2009, Zavil'gel'skii and Rastorguev 2009).

Pheromone inducible plasmids like tetracycline-resistance plasmid pCF10 contain a specific Prg/Pcf conjugation system. These respond only to CF10 pheromone levels through a quorum sensing mechanism to initiate conjugation (Dunny 2007). Pheromone inducible plasmids are ideal tools for genetic manipulation as they are highly efficient at DNA transfer (Staddon et al. 2006, Kohler et al. 2019). Enterococcal conjugative plasmids can additionally mobilise other plasmids in the cell (even non-conjugative plasmids) and lead to the formation of chimeric plasmids through recombination (Di Sante et al. 2017). This can be an issue when working with a clinical strain which contains multiple plasmids (Shan et al. 2022). Enterococcal conjugative plasmids containing *hyl*_Efm_ have been shown to co-transfer with antibiotic resistance regions such as *vanA* and may play a role in the dissemination of antibiotic resistance elements in clinical *E. faecium* strains (Arias et al. 2009).

Another important plasmid family for *E. faecalis and E. faecium* are Inc18-containing plasmids, e.g. pIP501, which encode the *tra* operon (*traA, traN* and *traO)*, that controls the conjugative processes. TraA is a relaxase that negatively regulates *tra-*operon transcription. TraN has a multi-layered function and works as a repressor of the conjugative system by blocking the *tra*-operon promoter (P_tra_) and also represses the *TraNO* promoter (P_traNO_). TraN is postulated to regulate itself as well as production of TraO, the surface adhesion required for cell-to-cell conjugation (Kohler et al. 2019). Conjugation protocols for enterococci are based on the single stationary phase timepoint protocol described by Simonsen *et al*(Simonsen et al. 1990). The recipient cell is resistant to an antibiotic marker not produced by the donor and therefore allowing for the direct selection of transconjugants upon plasmid transfer. Clinical *E. faecalis* and *E. faecium* strains are normally spoilt for choice due to the presence of multiple antibiotic markers. Kristich et al. constructed a spectinomycin-resistant *E. faecalis* donor strain for RepA-dependent conjugative plasmid delivery (pWV01 replicon plasmids), called CK111 (Kristich et al. 2007b). The chromosomally integrated pWV01 *repA* gene is under the control of the lactococcal P_23_ promoter, which was inserted into the *upp* locus, making the donor strain resistant to 5-flourouracil (5-FU). *E. faecalis* CK111 would be ideal for use in plasmid transfer experiments into clinical *E. faecium* strains.

## Bacteriophage transduction

Another mechanism of HGT is transduction, which is the introduction and potential integration of DNA into the host cell by a bacteriophage or virus like particles (VLPs) (Kleiner et al. 2020). Temperate bacteriophage enter the cell and multiply without disrupting the cells, while lytic phages multiply and cause cell lysis. Temperate phages can integrate at specific *attB* site(s) in the host genome and enter a lysogenic phase of phage dormancy. Integrated phage can excise from the chromosome and replicate upon sensing appropriate signal(s) (Mazaheri Nezhad Fard et al. 2011, Johnson et al. 2021).

Enterococcal bacteriophages are heavily implicated in HGT of antimicrobial and virulence genes in bacterial populations. Clinical MDR *E. faecium* have twice as many MGE-associated genes as non-clinical strains, which have most likely been acquired via MGE such as bacteriophages or plasmids (Buultjens et al. 2017, Johnson et al. 2021). *E. faecalis* VRE isolate V583 contains seven prophage-like elements that could contribute to the virulence and fitness of this isolate (Johnson et al. 2021). However, even though MDR *E. faecium* strains pose a threat to healthcare little is known about the role bacteriophage play in the population (eg. acquisition of novel virulence, immune evasion or antibiotic resistance genes), due to research being hindered by issues with current genetic manipulation techniques (Mazaheri Nezhad Fard et al. 2011, Johnson et al. 2021).

Generalised transduction is the non-specific accidental packaging of DNA into a bacteriophage during replication, which can facilitate the transfer of plasmids or chromosomal loci from one bacteria to another (Chiang et al. 2019). The application of generalized DNA transduction was first documented in *Salmonella* with phage P22, which is a *pac-*type phage which recognise *pac* sites on the bacterial or plasmid DNA to start DNA packing into the phage (Zinder and Lederberg 1952, Chiang et al. 2019). Lysogenic phages of *E. faecalis* have been identified which are capable of generalised transduction, which potentially could be applied to *E. faecium* (Yasmin et al. 2010).

Specialised transduction occurs through the accidental transfer of sequence specific DNA in proximity to the chromosomal phage attachment site and subsequent transfer. An example of a specialized transducing phage is *E. coli* lambda, which can also co-transduce with *gal* locus, at its integration site (Rédei 2008). In *Mycobacterium tuberculous* specialised transduction has been repurposed to facilitate a highly efficient one step allelic exchange system (Jacobs 2014, Jain et al. 2014). A similar approach could be modified and used to alter gene expression or construct targeted mutations in *E. faecium*. The presence of lysogenic bacteriophage could be identified through mitomycin C treatment of *E. faecium* strains with their ability to conduct generalised or specialised transduction assayed. Additionally, phage integrase genes could be repurposed for the construction of single copy phage integrase vectors (discussed below).

## Electroporation

**Figure 2.**
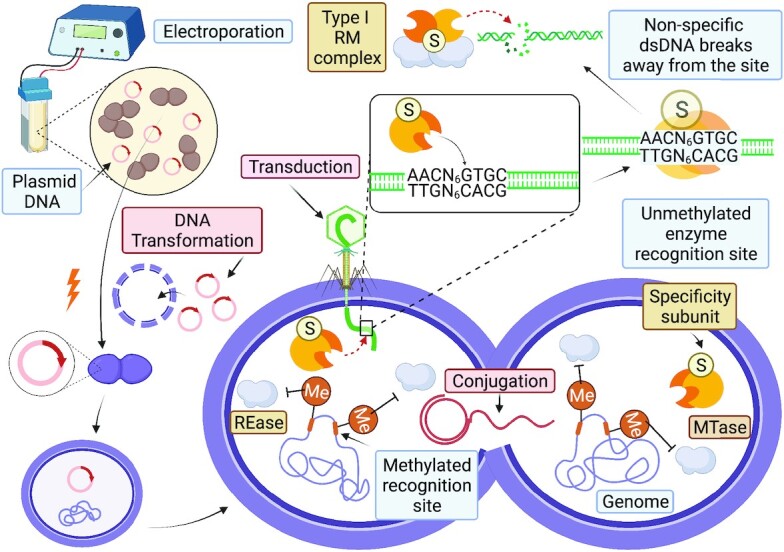
Mechanisms of DNA uptake in enterococci. A schematic diagram of DNA uptake in enterococci via transformation of plasmid DNA using electroporation and subsequent conjugation of plasmid DNA to another cell and transduction of phage DNA into the cell. Shown is a type I restriction modification system, a (HsdS) specificity protein and (HsdM) MTase complex (HsdM_2_HsdS_1_) has adenine methylated at the HsdS recognition sequences on the host genome to inhibit the generation of the RM complex (HsdM_2_HsdS_1_HsdR_2_). The foreign phage DNA is not methylated and is targeted by the HsdM_2_HsdS_1_, which binds the unmethylated recognition site, and then the HsdR_2_ yielding the RM complex, which then non-specifically induces dsDNA breaks away from the recognition site by collision (Phillips et al. 2019b). Created with BioRender.com.

Enterococcal species are not known to be naturally competent necessitating the use of other methods to introduce foreign DNA into the cell (Fig. 2), with electroporation the method of choice. A common transformation method is the use of stationary phase cells, (which exhibit a 10-fold improvement in transformation efficiency over exponential phase cells), repeated washes in 10% glycerol to remove salts and storage at -70°C (McIntyre and Harlander 1989). They are then electroporated with *E. coli* isolated plasmid DNA and recovered in nutrient rich media prior to plating on antibiotic selective agar. A variable transformation efficiency was observed, with between 10^0^and 10^5^ per µg of DNA with a range of enterococcal species (5 × 10^4^ per ug for *E. faecalis* JH2-2) (Friesenegger et al. 1991). However, over half of the enterococcal strains tested were not transformable by this approach. This study tested one *E. faecium* strain ATCC 9790, which has since been reclassified as *E. hirae* (Friesenegger et al. 1991, Kristich et al. 2007, Gaechter et al. 2012).

Further optimisation of the protocol for *E. faecalis* has included supplementation of the media with glycine to weaken the peptidoglycan in the presence of sucrose to act as an osmotic stabiliser (Shepard and Gilmore 1995). Post growth, all reagents added to the cells are kept on ice (wash buffers, plasmid and cuvettes) as it was shown to increase electroporation efficiency (Shepard and Gilmore 1995). Addition of ice-cold broth post electroporation and incubation on ice to delay the closure of membrane pores improved DNA uptake. Between 150 ng to 1000 ng of plasmid was recommended for transformation as the electroporation efficiencies were shown to decrease over 1 µg, while in *S. aureus* concentrations up to 10 µg can improve the total number of transformants obtained (Shepard and Gilmore 1995, Monk et al. 2015). The glycine method (Shepard and Gilmore 1995) yielded 1 × 10^6^ per µg of DNA for *E. faecalis* strain JH2-2, which is a 20-fold improvement over previous efforts (Friesenegger et al. 1991).

For *E. faecalis* the addition of lysozyme to log phase cells (OD_600_ 0.5–1) to weaken the peptidoglycan improved DNA uptake (Bae et al. 2002). A similar lysozyme or combined with mutanolysin approach was shown to work for *E. faecium* (Huo et al. 2019, Chen et al. 2021). It has also been observed that a protocol that works for one strain might not be optimal for another, therefore protocols should be tested empirically (Chen et al. 2021) Methods to overcome the poor electroporation efficiency in *E. faecium* will allow streamlined introduction of plasmid, potentially by using methylated DNA to bypass the RM systems found in *E. faecium* and *E. faecalis*. While optimised electroporation parameters are essential to maximise plasmid transfer, such conditions will not impact any endogenous enzymatic restriction of plasmid DNA. Where restriction of DNA is a barrier, application of a plasmid artificial modification approach (as described above for *S. aureus* (Monk et al. 2015)) through the expression of methylases in *E. coli* to mimic the adenine and/or cytosine methylation profile of the target strain can help unlock the genetics of hard to transform bacteria (Purdy et al. 2002, O'Connell Motherway et al. 2009, Johnston et al. 2019, Lee et al. 2019, Riley et al. 2019) and could be beneficial for some enterococci.

## Transformation via protoplast formation

Protoplast transformation of enterococci has been largely superseded with the advent of electroporation. Reversible generation of *E. faecalis* protoplasts has been extensively detailed (Wirth et al. 1986). This study optimised the protoplast transformation method using the shuttle vector pAM401 and generated 1 × 10^6^ transformants per µg of plasmid. Cells were grown overnight in Todd-Hewitt broth (THB) with 2%-4% glycine, then sub-cultured in the same media to early log phase, harvested and stored in 50:50 THB: glycerol at -70°C. Thawed cells were enzymatically digested with lysozyme to remove the peptidoglycan for 60–120 minutes in the presence of BSA. Plasmid was added to the protoplasts in buffer containing 40% polyethylene glycol, mixed, incubated on ice and then at 37°C. Cells were recovered in THB with osmotic support at 37°C and plated onto selective media for 2 to 3 days (Wirth et al. 1986). The protoplast transformation method was further optimised by Demuth et al to identify the ideal glycine concentration and lysozyme incubation time (Demuth et al. 1989).

## Genetic manipulation techniques in enterococci

The ability to readily and reproducibly introduce to DNA into enterococci opens the door to rapid and sophisticated genetic techniques. Techniques such as Mu transposon mutagenesis, construction of genomic, surface display and CRISPRi libraries, bypassing *E. coli* for the cloning and expression of toxic genes, phage integrase vectors and rapid allelic exchange all require highly competent cells (greater than 10^5^ CFU transformants)(Poquet et al. 1998, Pajunen et al. 2005, Monk et al. 2008, Monk et al. 2010, Bose et al. 2012, Jiang et al. 2020, Monk and Stinear 2021).

## Transposon mutagenesis

Random transposon mutagenesis and construction of transposon mutant libraries is a means to identify genes that are essential or conditionally essential. If a transposon insertion is in an essential gene the cell will die, therefore no transposon mutants will be found. However, insertions into some non-essential genes will lead to a growth-impaired phenotype under certain conditions. The location of the insertion can be determined by DNA sequencing. For example, as a platform for studying the genetic basis of antibiotic resistance, biofilm formation and fitness of *E. faecalis* in the human GI tract among other phenotypes, an ordered transposon library was created in *E. faecalis* OG1RF, a plasmid-free strain amenable to genetic manipulation (Dale et al. 2018).

High density transposon mutant libraries have also been generated in two *E. faecium* isolates using either microarray (isolate E1162) (Zhang et al. 2012) or transposon sequencing (Tn-seq) (isolate E745) to determine insertion sites (Zhang et al. 2017). Tn-seq involves a high-throughput DNA sequencing approach to identify genes that are essential, conditionally essential or exhibit reduced fitness under certain growth conditions *e.g*. human serum. (van Opijnen and Camilli 2013, Zhang et al. 2017, Cain et al. 2020).

## Allelic exchange

Allelic exchange involves the replacement of a target DNA region through homologous recombination, which can introduce a single base change, heterologous gene(s) or gene deletion. Often temperature sensitive plasmids are used to transfer the deletion construct (region of homology surrounding the desired mutation) into the target strain. First, a single crossover event is selected at a non-permissive temperature for plasmid replication (in the presence of antibiotic selection for the plasmid) through integration either upstream or downstream region of the target loci. A shift to a permissive temperature for plasmid replication (without antibiotic selection) leads to plasmid excision from the chromosome and subsequent loss from the cell. If the second crossover event is in the same region as integration, the strain will remain wild type, but if it occurs through the opposite region, it will generate a mutant. Colony PCR can be used to differentiate the wild type from mutant. This process can be very time consuming with the wild-type reversion often dominant (Monk and Foster 2012, Lehman et al. 2016).

The very low transformation efficiency or inability of clinical *E. faecium* isolates to take up DNA and lack of optimised techniques for allelic exchange has hampered their widespread investigation at a molecular level. To overcome these issues in *E. faecium*, Nallapareddy et al. developed an improved temperature-sensitive (ts) vector specifically for *E. faecium* called pTEX5500ts, which is based on the pWV01ts replicon (Nallapareddy et al.2006a). Still issues were encountered with clinical strains of *E. faecium*, but they were able to transform 5/11 strains tested (Nallapareddy et al.2006a). Using the same ts replicon, pIMAY-Z has been used to manipulate the *E. faecium* clinical ST796 isolate AUS0233 (Pidot et al. 2018). Even though AUS0233 has intrinsic beta-galactosidase activity, cells containing the plasmid can be discriminated from plasmid free strains on agar containing X-gal, facilitating rapid screening of plasmid excision.

For *E. faecium* a Cre-lox approach has also been developed, which allows the positive double crossover selection with an antibiotic cassette. A subsequent second round transformation with a temperature sensitive *cre*-expressing plasmid is used to excise the marker—leaving *frt* site scars (Zhang et al. 2012). In *E. faecalis*, a positive selection step to enrich for the second crossover was developed through the toxicity of 5-FU mediated by the *upp* encoded uracil phosphoribosyl transferase (Kristich et al. 2005). Upp is required for the pyrimidine salvage pathway. However, application of this approach requires the deletion of the chromosomally encoded *upp* which may subsequently impact on multiple cellular processes. A second approach for positive selection, which has been applied to both *E. faecalis* and *E. faecium* is the PheS* system (Kristich et al. 2007b, Panesso et al. 2011). The cell become exquisitely sensitive to the *p*-chloro-phenylalanine (*p*-Cl-Phe) in the presence of the mutated phenylalanine tRNA synthetase allele (denoted as PheS*). *p*-Cl-Phe is added to the broth to stimulate plasmid excision prior to agar selection. Additional benefit of the approach is that the allelic exchange plasmid (based on the suicide vector pORI280 (Leenhouts et al. 1998) containing constitutive LacZ, *oriT* from pCF10 and PheS*-pCJK47 or pHOU1) can be conjugated from a permissive donor strain CK111 (*E. faecalis* OG1Sp *upp4::P_23_-repA* containing pCF10-101) to either *E. faecalis* or *E. faecium* (Kristich et al. 2007b, Panesso et al. 2011).

The *Streptococcus pyogenes* Cas9 endonuclease has recently been applied to the targeted rapid allelic exchange in *E. faecium* with positive selection (de Maat et al. 2019), and yielded targeted mutants in three weeks. In an alternate approach, the *S. pyogenes* Cas9 positive counter-selection has been combined with a single stranded DNA binding protein (RecT recombinase). Cas9 counterselection and enhanced recombination mediated by RecT with either ssDNA or dsDNA template has increased the speed (under two weeks) and precision for the construction of substitutions, insertions and deletions (Chen et al. 2021). However, the size of deletions that can be constructed with ssDNA is currently limited as has been documented in other bacteria Gram-positive bacteria (Oh and van Pijkeren 2014, Penewit et al. 2018). Additionally, dsDNA insertions required a positive selection through the introduction of antibiotic marker. Recently, a new gene editing system for *E. faecium* was developed using CRISPR-Cas12a (also called Cpf1). The gene-editing Cas12a system only requires a CRISPR RNA (crRNA) for DNA targeting compared to the two RNA molecules required for Cas9 systems. The T-rich (5′-TTTV-3′) protospacer adjacent motif (PAM) sequence for Cas12a is common in low-GC bacteria, such as *E. faecium*, making this an ideal tool for targeted genetic manipulation. This study validated that the single plasmid Cas12a system could generate clean deletions or insertions within two weeks and is a breakthrough in optimising CRISPR for the genetic manipulation of *E. faecium* (Chua and Collins 2022). For the Cas9 system, two rounds of cloning (gRNA and homology arms) into a single plasmid are required, while in the Cas12a system this has been streamlined into one round and applies an *in vivo* cloning approach. Broader application of the Cas9 and Cas12a systems for genome editing will allow the assessment of mutation specificity and the potential for off-target, second site mutations of the respective systems.

These studies highlight the utility of CRISPR-Cas9 and Cas12a machinery as an efficient and timesaving method for genetic manipulation in *E. faecium*. In situations where gene inactivation is not possible (essential genes) or required (rapid candidate screening) CRISPR interference (CRISPRi) can be applied. CRISPRi uses a catalytically inactive Cas9 (dCas9–unable to cause DNA breaks) that can bind to target DNA and sterically block transcription, which allows for titratable control of target gene expression. A two-plasmid CRISPRi system for *E. faecalis* has been constructed comprising nisin-inducible (1) dCas9 and (2) guide RNA (Afonina et al. 2020). Analogous to transposon mutagenesis, the production of saturated CRISPRi libraries has been realised through the application of CRISPR adaptation-mediated library manufacturing *in vivo*. This approach greatly simplifies the construction and reduces the cost of CRISPR guides. These libraries can examine the partial suppression of essential genes and highlight genes involved in resistance pathways that have been overlooked by less complex libraries (Jiang et al. 2020). This approach should also be applicable to enterococci.

Allelic exchange is currently the ‘gold standard’ for genome editing in enterococci and has been used widely for many years (Kristich et al. 2005). However, there is still room for improvement such as the speed for mutant generation *e.g*. lack of positive selection and poor resolution of blue/white screening due to inherent beta-galactosidase activity (Kristich et al. 2005, Zhang et al. 2012, de Maat et al. 2019). The application of CRISPR technology to genome editing in enterococci is a recent development in the field (de Maat et al. 2019, Chen et al. 2021, Chua and Collins 2022). The advances in the speed of mutant generation (within 2–3 weeks), positive selection, choice of either 5′-NGG-3′ (Cas9) or 5′-TTTV-3′ (Cas12a) PAM, is a major step forward and could revolutionise genetic manipulation of enterococci.

## Complementation

Once a gene is deleted/modified a mutant is created that may exhibit a phenotype different from the wild type. Genetic complementation is essential for validation of the genotype/phenotype. Complementation involves the re-introduction of a wild type gene into the mutant to confirm the relationship. The original locus can be restored by ‘reverse’ allelic exchange with the complemented strain marked with a silent restriction site (Karnik et al. 2013). Genes can also be introduced into neutral loci on the chromosome, meaning a site that is not essential and will not generate a fitness cost if altered. Ideally the copy number and expression profile of the complemented gene will mimic the wild-type strain. It is important to whole genome sequence the mutant and complemented strain to ensure that no secondary mutations were generated that could give an altered phenotype. (Karnik et al. 2013, Azizoglu et al. 2014, Arras et al. 2015, Monk et al. 2017, Monk and Stinear 2021).

Shuttle vectors are also a common approach to complement a mutation, as shown for the restoration of cell wall localisation in a complemented *E. faecalis* ∆*srtA* mutant (Kristich et al. 2005). While single copy complementation to minimise gene dosage effects is often preferred but can be a more time-consuming option. DebRoy et al. (Debroy et al. 2012) identified a conserved, neutral site (between convergent genes in *E. faecalis*) for gene insertion through allelic exchange using the pCJK47 counterselection system (described above). This approach has the added advantage of not requiring antibiotic selection to maintain the gene of interest (Debroy et al. 2012).

Another approach are non-replicative phage integrase vectors that lead to single-copy integration at a specific bacterial attachment site *(attB*) on the chromosome mediated by the integrase/*attP* on the vector. Examples of these include TP901-1 of *Lactococcus lactis* and the PSA listeriophage in *Listeria monocytogenes*(Monk et al. 2008, Koko *et al*. 2019). One phage integrase vector has been successfully developed for *E. faecalis* (Yang et al. 2002).

Inducible expression of the complemented gene enables the modulation of gene expression. Systems that have been applied to enterococci include IPTG (Chen et al. 2021), rhamnose (Kristich et al. 2007a) pheromone (Weaver et al. 2017) or agmatine inducible promoters (Linares et al. 2014). For enterococci the nisin-controlled-expression systems (NICE) has also been widely applied (Afonina et al. 2020). The *nisRK* two component system encoded on the plasmid activates transcription of the P*nisA* promoter upon sensing the addition of nisin to the media (Bryan et al. 2000). The NICE system is commonly used in enterococci genetics because of the wide dynamic range achieved with a low concentration of nisin that does not inhibit bacterial growth (Eichenbaum et al. 1998). A recent adaption of the NICE system through the construction of a chimeric histidine kinase (NarQ; *E. coli* extracellular and transmembrane domains/NisK; cytoplasmic region) to change the signal sensed from nisin to nitrate. These modifications yielded a novel nitrate inducible expression system (Mascari et al. 2022). Other well characterised titratable systems such as tetracycline (Bertram et al. 2022) or cumate (Seo and Schmidt-Dannert 2019) could potentially be applied enterococci through the development of enterococcal specific promoters. For the inducible expression systems available for enterococci, there is no standardised comparison of the dynamic range of in the presence of inducer or repression achieved without induction in a single strain background. The application of inducible promoters is ideal for genetic complementation, especially in situations where toxicity is observed or to investigate expression over a dynamic range.

## Conclusions and Future Perspectives

In this article we have discussed the current methods and barriers to the genetic manipulation of enterococci. Where RM-systems in enterococci are identified the use of methylation modified plasmids would likely increase transformation efficiency and permit genetic manipulation of diverse clinical *E. faecium* isolates. Alternatively, the identification of transformable, clinically relevant sequence types (which lack enzymatic and have physical barriers which can be bypassed) would accelerate our ability to conduct genetic studies. Overall, it is known that *E. faecalis* is easier to transform and manipulate than *E. faecium*, as the former species has on average higher transformation efficiencies (Ike et al. 1998, Nallapareddy et al. 2006a). Tools and techniques including electroporation, transformation, transposon mutagenesis, and allelic exchange protocols are normally taken from *E. faecalis* and applied to *E. faecium* with variable efficiency (Wirth et al. 1986, Cruz-Rodz and Gilmore 1990, Kristich et al. 2005, Kristich et al. 2008). By using the research and techniques reviewed here to create specific protocols for *E. faecium* it should be possible to apply to powerful modern methods of genetic engineering such as transposon mutagenesis and CRISPR systems, to streamline and optimise mutant generation. The ability to genetically manipulate clinically relevant strains will permit scientists to answer more in-depth questions about the pathogenesis of enterococci, especially with regard to healthcare environments.
